# Type IX secretion system PorM and gliding machinery GldM form arches spanning the periplasmic space

**DOI:** 10.1038/s41467-017-02784-7

**Published:** 2018-01-30

**Authors:** Philippe Leone, Jennifer Roche, Maxence S. Vincent, Quang Hieu Tran, Aline Desmyter, Eric Cascales, Christine Kellenberger, Christian Cambillau, Alain Roussel

**Affiliations:** 1 Architecture et Fonction des Macromolécules Biologiques, Aix-Marseille Université, UMR 7257, 163 Avenue de Luminy, Case 932, 13009 Marseille, France; 2 Architecture et Fonction des Macromolécules Biologiques, Centre National de la Recherche Scientifique, UMR 7257, 163 Avenue de Luminy, Case 932, 13009 Marseille, France; 3 Laboratoire d’Ingénierie des Systèmes Macromoléculaires, Institut de Microbiologie de la Méditerranée, Centre National de la Recherche Scientifique (UMR7255), Aix-Marseille Université, 31 Chemin Joseph Aiguier, 13402 Marseille Cedex 20, France

## Abstract

Type IX secretion system (T9SS), exclusively present in the *Bacteroidetes* phylum, has been studied mainly in *Flavobacterium johnsoniae* and *Porphyromonas gingivalis*. Among the 18 genes, essential for T9SS function, a group of four, *porK-N* (*P. gingivalis*) or *gldK-N* (*F. johnsoniae*) belongs to a co-transcribed operon that expresses the T9SS core membrane complex. The central component of this complex, PorM (or GldM), is anchored in the inner membrane by a trans-membrane helix and interacts through the outer membrane PorK-N complex. There is a complete lack of available atomic structures for any component of T9SS, including the PorKLMN complex. Here we report the crystal structure of the GldM and PorM periplasmic domains. Dimeric GldM and PorM, each contain four domains of ~180-Å length that span most of the periplasmic space. These and previously reported results allow us to propose a model of the T9SS core membrane complex as well as its functional behavior.

## Abstract (web summary)

No structural data for the bacterial type IX secretion system (T9SS) are available so far. Here, the authors present the crystal structures of the periplasmic domains from two major T9SS components PorM and GldM, which span most of the periplasmic space, and propose a putative model of the T9SS core membrane complex.

## Introduction

Bacteria, especially Gram-negative species, have assembled and evolved complex and specific cellular machines, known as secretion systems, to secrete proteins or DNA through the cell envelope into the surrounding medium or inside other cells^1–3^. In diderm bacteria, protein secretion occurs either as a one-step process, in which substrates are translocated directly from the cytoplasm to the external milieu, or as a two-step process, in which the substrates first cross the inner membrane (IM) into the periplasm using the Sec, Tat, or holins pathways and then cross the outer membrane (OM) through a specialized translocon^2^. After secretion, the substrates might stay attached to the OM surface, be released into the extracellular milieu, or be injected into a target cell^2^. The type IX secretion system^1,4–6^ (T9SS) uses a two-step process. Depending on the bacterial strain, the T9SS confers very distinct functions. In *F. johnsoniae*, the T9SS contributes to gliding motility by secreting SprB, a cell-surface adhesin that is required for movement on solid surfaces^7^. *P. gingivalis*, a non-motile bacterium, is a human oral pathogen and a major causative agent of periodontitis, as its T9SS secretes potent proteolytic enzymes called gingipains^8^ that degrade host cell tissues and interfere with innate host defense mechanisms^9^. To date, 18 genes have been identified as essential for T9SS function in *P. gingivalis*^10^. Among them are a group of five genes, *porP–porK–porL–porM–porN*, which belong to a co-transcribed operon^3^. The last four genes have orthologues in the *F. johnsoniae* genome, *gldK–gldL–gldM–gldN*, together with other extra orthologues of *porM*^11^. PorK, PorL, PorM, and PorN, assemble as a >1.4 MDa trans-membrane complex^1^. PorK (or GldK) is a lipoprotein anchored to the OM that interacts with the periplasmic protein PorN^7,12^. PorL and PorM (GldL and GldM) are IM proteins that interact via their trans-membrane segments. The core of PorL resides in the cytoplasm, whereas PorM, similar to GldM, has a long periplasmic domain^3^. PorM interacts with both PorK and PorN complex, and therefore spans the entire periplasm by being anchored in the IM and interacting with the OM complex^3^. PorM (or GldM) is therefore a central structural component of the T9SS and an interesting target for structure and function studies. Here, we present the atomic structures of the periplasmic domains of both PorM and GldM, that exhibit 22% amino-acid identity, and provide information regarding the contribution of each domain for interaction with PorK and PorN.

## Results

### Three-dimensional structure of GldM

The GldM (accession number GI: 58531935) and PorM periplasmic domains (GI: 188595218) (GldMp, PorMp) were cloned from residues 36–513 and 36–516, respectively. GldMp crystallized readily, and its structure was solved using the Se edge of a SeMet derivative for phasing (Table 1). One molecule was present in the asymmetric unit, but strong contacts exist with a symmetry-related protein in the crystal. The assembly of GldMp as a dimer was confirmed because domain swapping and tight locking to the symmetry-related dimer were observed, which have been previously demonstrated for GldM in solution, as assessed by size exclusion chromatography (SEC).

**Table 1:** Data collection phasing and refinement statistics

	GldMp	GldMp SeMet-SAD	PorMp_224_		PorMp_224_SeMetMAD		PorMp_315_/nb130	PorM_Nt_/nb01
Data collection								
Space group	P6_4_22	P6_4_22	P4_3_2_1_2		P4_3_2_1_2		P1	P2_1_
Cell dimensions								
* a*, *b*, *c* (Å)	71.4, 71.4, 426.9	71.40, 71.40, 426.09	77.0, 77.0, 228.6		77.4, 77.4, 226.9		55.2, 77.2, 156.3	80.3, 99.9, 80.3
* α*, *β*, *γ* (°)	90, 90, 120	90, 90, 120	90, 90, 90		90, 90, 90		90.2, 91.7, 97.2	90, 93.8, 90
				*Peak*	*Inflection*	*Remote*		
Wavelength	0.9677	0.97908	0.97888	0.979109	0.979336	0.976256		
Resolution (Å)	46.7–2.0 (2.11–2.0)	47.3–2.4 (2.53–2.4)	40–2.85 (3.0–2.85)	50–3.1 (3.27–3.1)	50–3.1 (3.27–3.1)	50–3.1 (3.27–3.1)	40–2.1 (2.21–2.1)	42.5–2.4 (2.53–2.4)
* R*_merge_	0.120 (2.566)	0.146 (3.212)	0.076 (0.764)	0.091 (0.710)	0.093 (0.827)	0.075 (0.569)	0.053 (0.625)	0.052 (0.865)
* I*/*σI*	10.1 (1.2)	13.6 (0.9)	14.8 (2.2)	13.2 (2.8)	13.1 (2.5)	9.2 (9.8)	12.5 (2.0)	11.6 (1.0)
Completeness (%)	100.0 (100.0)	100.0 (99.9)	99.9 (100.0)	98.8 (98.8)	98.8 (98.7)	99.0 (98.8)	97.6 (96.8)	98.3 (96.7)
Redundancy	13.6 (14.4)	20.4 (21.7)	8.0 (8.0)	9.1 (9.7)	9.2 (9.8)	9.2 (9.8)	2.9 (2.9)	6.9 (6.8)
Refinement								
Resolution (Å)	43.4–2.0		39.4–2.85				38.3–2.1	40–2.4
No. of reflections	45,244		16,861				145,522	48,833
* R*_work_/*R*_free_	22.2/25.9		21.7/24.9				18.0/21.5	21.3/24.2
No. of atoms								
Protein	3534		4469				20,169	8569
Ligand/ion	24/—		—/4				—/—	—/—
Water	298		60				1236	159
* B*-factors								
Protein	69.6		109.5				53.4	109.4
Ligand/ion	89.4/—		—/167.1				—/—	—/—
Water	68.9		78.2				50.6	90.5
R.m.s deviations								
Bond lengths (Å)	0.009		0.01				0.01	0.009
Bond angles (°)	1.16		1.23				1.13	1.12

Values in parentheses are for highest-resolution shell

**Fig. 1:**
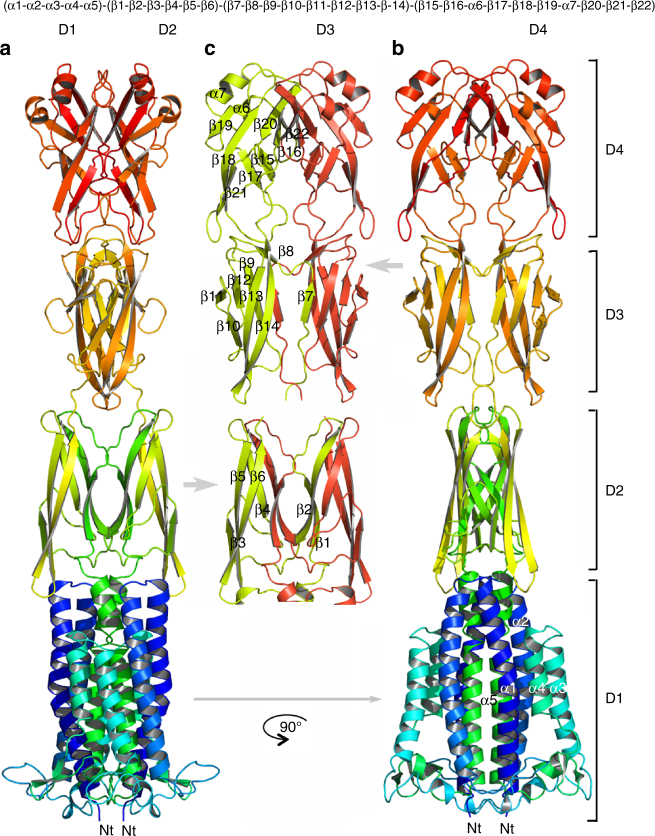
Crystal structure of GldMp from *Flavobacterium johnsoniae*. **a** Ribbon view of the GldMp structure rainbow colored from the N-terminus (blue) to the C terminus (red). **b** The same representation, 90° from **a**. The four domains are labeled D1, D2, D3, and D4. **c** Domains are colored by polypeptide chain, yellow and red. Note the swapped β-strands in domains D2 (β1 and β2) and D3 (β7). Top, the secondary structure schematic

The GldMp dimer is elongated and straight with overall dimensions of ~180 Å × 50 Å × 35 Å (Fig. 1). The dimer structure contains four domains, D1–D4 (Fig. 1) with seven α-helices and 22 β-strands in the sequence (α1−α2−α3−α4−α5) (β1−β2−β3−β4−β5−β6) (β7−β8−β9−β10−β11−β12−β13−β14) (β15−β16−α6−β17−β18−β19−α7−β20−β21−β22).

Domain D1 (32-232) is formed by helices 1–5 in an up and down fold. The D1 domain dimers are packed together through helices α1. The D2 (233–320) and D3 (321–405) domains are exclusively formed of β-strands. Each D2 domain swaps its β-strands 1 and 2 with the other D2 domain, whereas D3 domains swap β-strand 7. The main plane of domain D3 is perpendicular to that of domain D2 (Fig. 1). The D4 domains (406–513) are not subject to domain swapping but are packed together in the dimer (Supplementary Table 1). The junctions between domains D1–D2 and D3–D4 are compact and thus prevent flexibility (Fig. 1; Supplementary Fig. 1). However, the D2–D3 junctions are less compact and suggest that some bending may occur in solution. Remarkably, with a 180 Å-extended conformation, GldM spans most of the periplasmic space, as the distances between the IM and OM associated with T3SS, T4SS, and T6SS have been found to be ~260^13^, ~170^14^, and 180 Å^15^, respectively.

### Three-dimensional structure of PorM

**Fig. 2:**
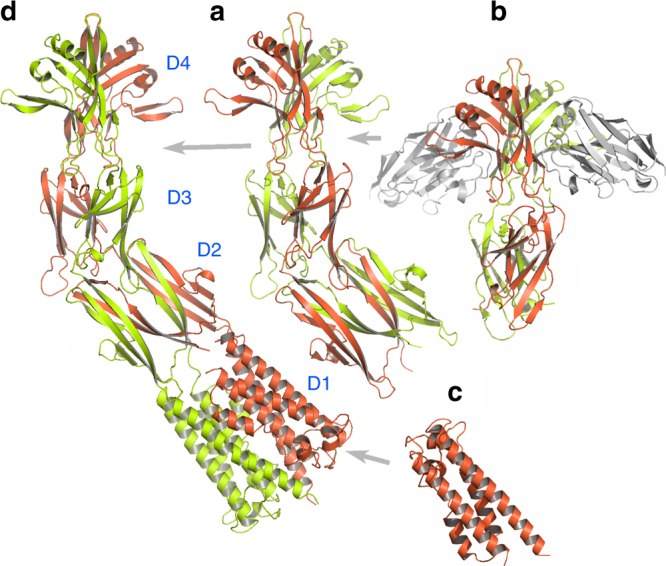
Crystal structure of PorMp from *Porphyromonas gingivalis*. **a** Ribbon view of the PorMp_224_ fragment structure (residues 224–516) and **b** of the PorMp_335_ fragment structure (residues 224–516) in complex with the nanobody nb130 colored by polypeptide chain (yellow and red, gray for nb130). **c** Ribbon view of the PorMp_nt_ fragment (nanobody not shown). **d** Ribbon view of a PorMp model obtained by aligning the N-terminal D1 domain and the D2 domain on the GldMp scaffold. The domains are numbered D1–D4

**Fig. 3:**
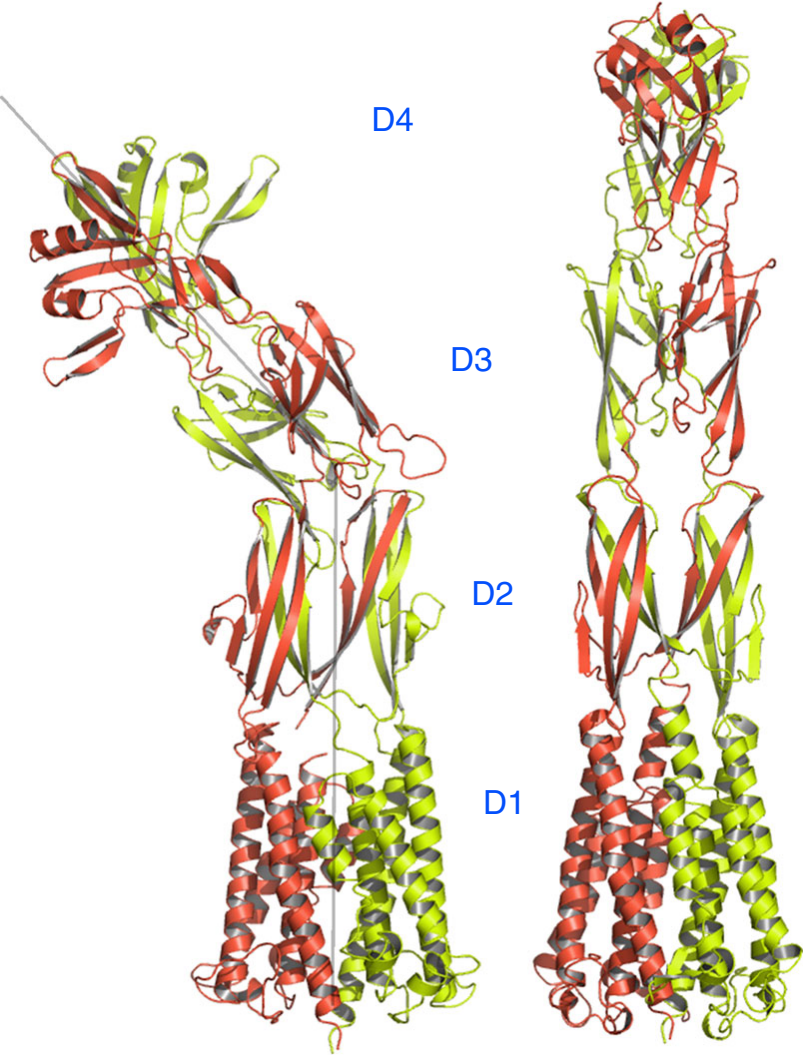
Comparison of GldMp and PorMp structures. Left, PorMp structure; right, GldMp structure (chain A and B are colored in green and red, respectively). Both structures have been aligned using their D1 and D2 domains. Domains are numbered D1–D4 from their N termini to C termini. Note the ~45° angle between D1–D2 and D3–D4 PorMp domains

The structural determination of PorMp was more tedious than that of GldMp. Full-length PorMp resisted all attempted crystallization assays. Trypsin cleavage experiments were therefore performed, and a defined fragment (residues 224–516, PorMp_224_) was purified and crystallized^16^ (Fig. 2a). Phasing was performed using SeMet-substituted PorMp_224_. Domains D2 and D3 could be traced fairly easily, but domain D4 was only partially constructed due to poor electron density map. In an attempt to stabilize this domain, we raised anti-PorMp llama antibodies and selected a nanobody (nb130) from the resulting library^17^ that bound to PorMp with high affinity (*K*_D_ = 4.5 nM)^16^. We cloned a PorMp fragment between residues 224–516, co-crystallized it with nb130, and determined the structure of the complex (Fig. 2b). Surprisingly, the crystallized structure contained only domains D3 and D4, meaning that domain D2 was cleaved by a protease during crystallization; thus, the resulting structure spans residues 315–516 (PorMp_315_). The D4 domain was easily traced in the electron density map because it was stabilized by nb130 binding, and it was introduced into the PorMp_224_ structure, generating a complete model. The resulting PorMp_224_ structure exhibits three domains that resemble GldMp domains D2–D4 (Figs. 1, 2b, and Fig. 3). Interestingly, PorMp domains D2 and D3 possess a domain-swapping motif identical to that of GldMp (Fig. 3; Supplementary Fig. 2). Finally, we cloned, crystallized, and determined the structure of the N-terminal domain of PorMp (residues 30–212) in complex with a nanobody (PorMp_Nt_) (Fig. 2c; nanobody not represented). Using the structure of GldMp, we could assemble PorMp_Nt_ and PorMp_224_ in a realistic model of full-length PorMp (Fig. 2d).

### Comparison between GldM and PorM

Taken individually, the four domains of PorMp superimpose well with those of GldMp, with root mean square deviation values ranging from 1.6 to 3.5 Å (Supplementary Table 2; Supplementary Fig. 2). Domains D3 and D4 of PorMp_224_ and PorMp_315_ share the same straight topology as those of GldM, whereas domain D2 is bent with respect to D3–D4 at an angle of ~45° because of the convolution of two rotations and a sliding of D3 monomers (Fig. 3; Supplementary Fig. 3).

Surprisingly, PorMp_Nt_, the D1 domain of PorM, is missing the first helix (residues 30–69), which was probably cleaved during crystallization (Fig. 2c; Supplementary Fig. 2). Another difference between the D1 domains of GldMp and PorMp is the organization of dimer packing. In GldMp, both D1 domains are packed side by side using their α1 helices; in PorMp_Nt_, the four monomers in the asymmetric unit do not pack together, probably because the α1 helix that forms the D1 domain interface in GldMp is absent in this structure (Fig. 1). Finally, a complete domain can be modeled by assembling the various fragments using the GldMp scaffold: PorMp_315_ was structurally aligned with PorMp_224_, the PorMp_224_ D2 domain was aligned with the GldMp D2 domain, and the PorMp_Nt_ D1 domain was aligned with the GldMp D1 domain, together with the modeling of the first PorMp_Nt_ helix using α1 from GldMp D1 (Fig. 2d, e).

**Fig. 4:**
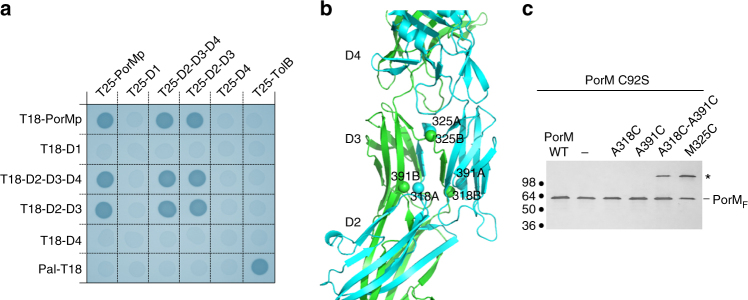
In vivo validation of the PorMp structure. **a** Bacterial two-hybrid analysis. BTH101 reporter cells carrying pairs of plasmids producing the indicated PorM fragments fused to the T18 or T25 domain of the *Bordetella* adenylate cyclase were spotted on X-Gal-IPTG reporter LB agar plates. The blue coloration of the colony reports interaction between the two partners. Controls include T18 and T25 fusions to TolB and Pal, two proteins that interact but unrelated to the T9SS. **b** Ribbon representation of a portion of PorM with the Cys mutants (tested in **c**) identified by spheres. **c** Disulfide-bond formation. Total membrane fractions from cells producing the wild-type (WT) FLAG-tagged PorM protein, and the indicated cysteine variants introduced into the cysteine-less C92S PorM, were subjected to 10%-acrylamide SDS-PAGE and immunodetected using the anti-FLAG antibody. The asterisk on right indicates bands corresponding to a PorM dimer. Molecular weight markers are shown on right

An interesting feature of both GldM and PorM is their D2–D3 domain-swapping motifs. To test whether this domain swapping exists in vivo, we first performed bacterial two-hybrid (BACTH) experiments. We found that the D2–D3 construct oligomerizes, whereas the D1 and D4 isolated domains do not interact with themselves (Fig. 4a). On the basis of the structure of PorMp, we introduced cysteine residues at different positions within the D2 and D3 domains of the full-length PorM protein (Fig. 4b). SDS-PAGE analyses in absence of reducing agent demonstrated that residues Ala-318 from one monomer is at close distance from residue Ala-391 from the second monomer, whereas the Met-325 residues from two monomers face each other in the dimer (Fig. 4c). These results confirm that the domain swapping occurs in vivo, in the context of the full-length protein.

The most striking difference observed between GldMp and PorMp is their overall topology, which results from the PorMp kink between D2 and D3 (Fig. 3; Supplementary Fig. 3). This kink is the result of two rotations around a vertical axis and around a horizontal axis, as in a cardan mount (Supplementary Fig. 3). Of note, the PorMp D2–D3 bending movement can occur in the left or the right direction, leading to two non-superimposable structures. The observation of bending in a unique direction suggests that the two forms may equilibrate by exchange through a transient straight form resembling GldMp. During crystallization, the equilibrium would be displaced towards the form accommodated in the crystal.

**Fig. 5:**
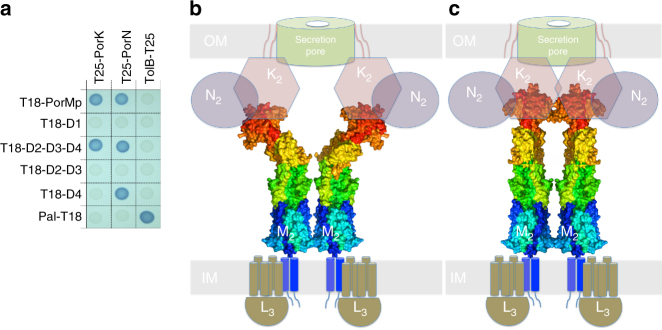
Topological and functional models of PorM and GldM core membrane complexes. **a** Bacterial two-hybrid analysis. BTH101 reporter cells carrying pairs of plasmids producing the indicated PorM fragments fused to T18, and PorK or PorN fused to T25 were spotted on X-Gal-IPTG reporter LB agar plates. The blue coloration of the colony reports interaction between the two partners. Controls include T18 and T25 fusions to TolB and Pal, two proteins that interact but unrelated to the T9SS. **b** Representation of an “open” topology (during secretion) and **c** of a “closed” topology (system at rest). The model shows PorM as in the crystal structure (**b**) and extended as in the GldM structure (**c**). The rest of the model collects data from previous reports^1,3,11^. K, L, M, and N schematically represent PorK, L, M, and N or GldK, L, M, and N

We previously reported that the periplasmic domain of PorM interacts with both PorK and PorN^7^. The contribution of PorM domains for contacting PorK and PorN was tested by BACTH. Our results show that the PorM–D4 domain is sufficient for interacting with PorN. By contrast, PorM interaction with PorK requires the D2–D3 and D4 domains, suggesting that either the three domains are required for interaction or that D2–D3-mediated dimerization of the D4 domain (monomeric in the isolated form) is necessary to properly interact with PorK (Fig. 5a).

### A putative multimer model of a complex

Using the data from Sato et al.^1^, proposing a mass slightly larger than 1.4 MDa of the (PorKLMN)_2_ complex^1^ and from Vincent et al.^3^, together with the structures reported here, we speculated on the possible architecture of the T9SS core machinery. We used Symmdock software^18^ to identify which part of PorM/GldM might fit together to form a multimer of dimers. Using the straight GldMp structure, both threefold and fourfold symmetry created associations involving mainly the D1 domain, and the rest of the structures exhibited a topology resembling that of tulip petals (Supplementary Fig. 4). This tulip shape is even more marked when PorMp is used, as its bending opens the D3–D4 arms to a larger degree. As a two-step secretion system, T9SS has to recruit effectors from the periplasm. To this end, the effectors must move through the PorM/GldM arches in a similar manner to how T2SS loads its cargo through the secretins^19,20^.

## Discussion

It has been reported by us^3^ and others^1^ that PorM binds to PorN. The *K*_D_ of the PorMp–PorN association, ~1 μM^3^, is comparable to that of the association between the TssJ lipoprotein with TssM (*K*_D_ = 2–4 μM^21^), an event that initiates T6SS core complex assembly at the OM^15^. In turn, PorN binds to the PorK lipoprotein; therefore, the T9SS PorK–PorN complex might represent a functional equivalent of the T6SS TssJ lipoprotein, with equivalent binding affinities for PorM or TssM. In the T6SS core complex, TssJ also binds the C-terminal D4 domain of TssM^15^.

Sato et al.^1^ isolated a core membrane complex of T9SS from *P. gingivalis* that was extracted using DDM. Analysis of this complex by western blot and SDS-PAGE revealed that it contains four components, PorK, PorL, PorM, and PorN, and that its mass is slightly larger than 1.4 MDa^1^. We recently reported that PorM, PorN, and PorK form homodimers, whereas PorL forms homotrimers^3^. Hence, the stoichiometry of the assembly is expected to be PorL_3_/PorM_2_/PorN_2_–PorK_2_, resulting in an overall mass of ~410 kDa. Therefore, three or four copies of the above-described assembly would be necessary to form the ~1.4 MDa isolated by Sato et al.^1^

In contrast, Gorasia et al.^12^ reported data that differ from those reported above. In their report, a *P. gingivalis* membrane fraction was purified leading to large rings that were analyzed with electron microscopy. These rings, which were attached to the membrane, measure ~50 nm in diameter (35 nm internally) and are formed of 32–36 1:1 PorK:PorN complexes. They did not observe the presence of PorM or PorL. These authors proposed that the native complex therefore contains 32–36 copies. The same rings were observed on the surface membrane of *P. gingivalis* mutants lacking *porL*, *porM*, and *porP*. Strangely enough, despite the strong interaction measured between PorM and PorN, PorM was not observed in the complex. Furthermore, a pore of 350 Å would be very difficult to occlude during non-secretion periods. We therefore suspect that the gigantic pore reported in Gorasia et al. might be due to the absence of PorM/PorL in the preparations, resulting either from purification or from the use of cells encoding a *porM*^−^ mutation. As often observed with protein-forming rings (e.g., phage portals^22^, RAD52, or viral nucleocapsids), ring stoichiometry might vary in the absence of controlling elements.

By assembling all of the available data, we propose a schematic model of the T9SS core complex and secretion-associated opening based on the topology of the PorL_3_/PorM_2_/PorN_2_–PorK_2_ moieties (Fig. 5b, c). Each PorMp dimer is anchored in the IM by its two helices, which interact with the three helices of the PorL trimer. Close to the OM, the PorM–D4 domain mediates contact with the PorN–PorK complex. The membrane-attached ring of PorN_2_/PorK_2_ should be associated with the secretion pore and may control its access by the effector. Interestingly, several possible candidates have been proposed controlling secretion, although no definitive arguments implicating a specific one have been made^5,10^. To note, Veith et al.^11^ proposed that a cascade of several OM components might be associated with the core machinery for the post-treatment of effectors and their eventual association with the OM^11^.

We speculate that the hinge between D2 and D3 may have a role in PorN/PorK opening, as it has been proposed that PorM is energized by the PorL trimer and that the two proteins form an energy transduction system for effector translocation^3,12,23^. The putative straight topology of PorM, resembling that of GldM, may therefore be associated with a closed state of the system. This state might be converted to the open form through a conformational change at the D2–D3 interface through PorL/PorM activation (Fig. 5b, c). Finally, we suggest that due to the structural similarity between PorM and GldM, both classical T9SS and Gld T9SS membrane core complexes might assemble and function in similar ways.

## Methods

### Protein production

The sequences corresponding to PorMp (residues 36–516), PorMp_315_ (residues 224–516), and PorMp_Nt_ (residues 44–217) were cloned into the pET28a+ derivative vector pLIC03 (Supplementary Table 3). The sequence corresponding to GldMp (residues 36–513) was amplified from *Flavobacterium johnsoniae* cDNA (ATCC17061, Leibniz Institute DSMZ) and was cloned into pLIC03 with the same protocol as for PorM constructs (Supplementary Table 3). PorMp, PorMp_315_, PorMp_Nt_, GldMp, and SeMet GldMp were produced in Rosetta *E. coli* cells and purified by nickel-affinity chromatography followed by SEC^16,17^.

### Production of PorM-specific llama nanobodies

The PorM-specific nanonbodies nb01 and nb130 were obtained and purified by standard methods^17^. In brief, a llama (*Lama glama*) was immunized with purified PorM_p_ (Ardèche-lamas France). PorM_p_ was injected subcutaneously four times at 1-week intervals using incomplete Freund’s adjuvant, followed by a fifth injection 2 weeks later. Blood samples were collected aseptically 5 days after the last boost. Lymphocytes were isolated from blood samples, and cDNA was synthesized from the acquired RNA using a reverse PCR protocol. A nanobody phage display library of ~10^9^ independent transformants was generated using the phagemid vector pHEN4^24,25^. Phage display selection and screening of specific nanobodies were performed as previously published^26^. After enrichment of antigen-specific clones by rounds of selection on solid-phase-coated antigen, PorM-specific nanobodies were identified, and the inserts of the corresponding pHEN4-derived plasmids were sequenced and cloned into the pHEN6 vector. *E. coli* WK6 cells carrying the pHEN6 derivatives were grown at 37 °C in terrific broth supplemented with 0.1% glucose and 100 μg/mL ampicillin to an optical density ~0.6–1.0 and the expression of the nanobodies was induced by the addition of 1 mM IPTG for 16 h at 28 °C. The periplasmic fraction containing the nanobodies was prepared using mild osmotic shock and the His-tagged nanobodies were immobilized on a 5-mL Ni-NTA column equilibrated in 50 mM Tris–HCl, pH 8, 300 mM NaCl, and 10 mM imidazole. Nanobodies were eluted in 250 mM imidazole and concentrated using the Amicon-technology (10-kDa cut-off) prior to loading on a HiLoad 16/60 Superdex 75 gel filtration column equilibrated in 20 mM Tris–HCl, pH 8, 50 mM NaCl.

### Crystallization and crystallographic processing

All crystallization trials were performed using the sitting-drop vapor-diffusion method at 293K in 96-well Greiner plates. Drops were prepared by mixing different volumes (100, 200, and 300 nL) of protein solution and 100 nL of precipitant solution, and were equilibrated against 150 μL reservoir volume.

Crystallization trials of PorMp_224_ and PorMp_224_ SeMet derivatives were performed with the PEGs Suite (Qiagen). After optimization, the final crystallization conditions were 0.02 M sodium acetate pH 4.8–5.8, 0.2 M zinc acetate, 15–25% (w/v) PEG3350, with a protein-to-well ratio of 3:1 (v/v). Crystallization trials of PorMp_315_/nb130 was performed with the 1/1 complex at 10 mg/mL with 0.2 M Ammonium citrate tribasic pH 7.0 20% (w/v) PEG3350 as precipitant. Crystallization trials of PorM_Nt_/nb01 was performed with the 1/1 complex at 10 mg/mL with 0.1 M Hepes pH 7.5, 0.2 M NaCl, and 25% (w/v) PEG3350 as precipitant. Crystals were briefly soaked in crystallization solution supplemented with 20% (v/v) propylene glycol before being flash-frozen in a nitrogen gas stream at 100K.

Native PorMp_224_ diffraction data were collected to 2.85 Å resolution on beamline ID23–1 at the European Synchrotron Research Facilities (ESRF, Grenoble, France), using a Pilatus detector. PorMp_224_ SeMet MAD data were collected to 3.1 Å on the same beamline. The data sets were integrated with XDS^27^ and were scaled with SCALA from CCP4 Suite v6.3.0^28^.

Complete data sets of PorMp_315_/nb130 and PorMp_Nt_/nb01 were collected at beamline PROXIMA 1 at SOLEIL, Paris, France. Data were integrated and scaled as for PorMp_224._ Data collection statistics are reported in Table 1.

Initial crystallization trials of GldMp and SeMet GldMp were performed by the sitting-drop vapor-diffusion method at 293K in 96-well Swissci plates using a Mosquito Crystal robot (TTP Labtech) with the following screens: Stura Footprint Screens (Molecular Dimensions), Structure Screen and Structure Screen 2 (Molecular Dimensions), PEGs Suite and PEGs II Suite (Qiagen), JCSG+ Suite (Qiagen), and Index (Hampton Research). Crystallization hit occurred in condition No. 8 of the Stura Footprint Screen #2 (0.1 M Hepes pH 7.5, 45%(w/v) PEG 600). After optimization, the final crystallization conditions were 0.1 M Hepes pH 7–8, 26–46% (w/v) PEG 600. Crystals were briefly soaked in crystallization solution supplemented with 10% (v/v) ethylene glycol and 10% (v/v) glycerol for native and SeMet GldMp, respectively. Native GldMp diffraction data were collected to 2 Å resolution on beamline ID30A-3 at the European Synchrotron Research Facility (ESRF), Grenoble, France. SeMet GldMp single-wavelength anomalous diffraction (SAD) data were collected to 2.4 Å resolution on beamline Proxima-1 at SOLEIL, Paris, France. A fluorescence scan was performed to determine the peak wavelength (0.97908 Å). The data sets were integrated with XDS^27^ and were scaled with SCALA from CCP4 Suite^28^. More technical details are provided elsewhere^16,17^. Data collection statistics are reported in Table 1.

### Structure determination

The structure of PorMp_224_ was solved by the multiple-wavelength anomalous diffraction (MAD) method using the SeMet PorMp_224_ data set at 3.1 Å resolution. Heavy-atom substructure determination, positional refinement, phase calculations, and solvent flattening were performed using autoSHARP^29^, SHARP^30^, and SOLOMON^31^. The partial model of SeMet PorMp_224_ was built using Turbo-Frodo^32^, and was subsequently used as model for molecular replacement with MOLREP^33^ to solve the structure of native PorMp_224_ at 2.85 Å.

The structure of the complex PorMp_315_/nb130 was solved by molecular replacement with MOLREP^33^ using the partial model of domains D3 and D4 of PorMp_224_, and the structure of nb130^17^ as models. The building of domain D4 of PorMp_224_ and PorMp_315_ was then completed manually with COOT^34^.

The structure of the complex PorMp_Nt_/nb01 was solved by combining molecular replacement with MOLREP^33^ using the structure of nb01^17^ as starting model, and several cycles of automatic building of PorMp_Nt_ in the extra density with BUCCANEER^35^ followed by refinement with autoBUSTER^36^. The building of PorMp_Nt_ was then completed manually with COOT^34^.

The structure of GldMp was solved by the SAD method using the SeMet GldMp data set collected at 2.4 Å. Heavy-atom substructure determination, positional refinement, phase calculations, and solvent flattening were performed using autoSHARP^29^, SHARP^30^, and SOLOMON^31^. The partial model of SeMet GldMp was automatically built with BUCANEER^35^, and was subsequently used as model for molecular replacement with MOLREP^33^ to solve the structure of native GldMp at 2 Å. The building of GldMp was then completed manually with COOT^34^.

Refinement, correction, and validation of the different structures were performed with autoBUSTER^36^, COOT^34^, and Molprobity^37^, respectively. More technical details are provided elsewhere^16,17^. Refinement statistics are reported in Table 1.

### Symmdock modelization

Symmdock software^18^ complex modeling was performed using the straight GldMp structure as input, with both threefold and fourfold symmetry. The two best solutions were found to be close together resulting in a tight-packed N-terminal domain. To note, Symmdock works by maximizing the contact surface between monomers and minimizing the steric clashes.

### Bacterial two-hybrid

PorM–D1, PorM–D2–D3–D4, PorM–D2–D3, and PorM–D4 domains fused to the T18 and T25 domains of the *Bordetella* adenylate cyclase have been engineered by restriction-free ligation. BACTH experiments have been performed as previously^3^. After introduction of the two plasmids producing the fusion proteins into the reporter BTH101 strain, plates were incubated at 30 °C for 48 h. Three independent colonies for each transformation were inoculated into 600 μL of LB medium supplemented with ampicillin, kanamycin, and IPTG (0.5 mM). After overnight growth at 30 °C, 10 μL of each culture were dropped onto LB plates supplemented with ampicillin, kanamycin, IPTG, and X-Gal, and incubated for 16 h at 30 °C. Controls include interaction assays with TolB/Pal, or MalF/MalG, two protein pairs unrelated to the T9SS. The experiments were done at least in triplicate and a representative result is shown.

### In vivo disulfide-bond formation

Cysteine codons were introduced by Quick change site-directed mutagenesis into the plasmid encoding the C92S variant of FLAG-tagged PorM^7^. After gene induction, cells were lysed, and the total membrane fractions obtained after ultracentrifugation were subjected to 10%-acrylamide SDS-PAGE, transfer to nitrocellulose, and immunodetection using monoclonal anti-FLAG antibody. Figure 4c uncropped gel is provided as Supplementary Fig. 5.

### Data availability

Structures of PorMp_224_, complexes PorMp_Nt_/nb01 and PorMp_315_/nb130, and GldMp were deposited in the Protein Data Bank (PDB) under accession numbers 6EY5, 6EY0, 6EY6, and 6EY4, respectively. Other data are available from the corresponding authors upon reasonable request.

## Electronic supplementary material

Supplementary Information
