# Supplementary material for: Type IX secretion system PorM and gliding machinery GldM form arches spanning the periplasmic space
Source: Nat Commun. 2018 Jan 30;9:429. doi: 10.1038/s41467-017-02784-7 (PMC5790014; doi:10.1038/s41467-017-02784-7)
Supplement: Supplementary file 1 — Supplementary Information [file 41467_2017_2784_MOESM1_ESM.pdf]

Type IX secretion system PorM and gliding machinery GldM form extended arches spanning the periplasmic space.

Leone *et al.*

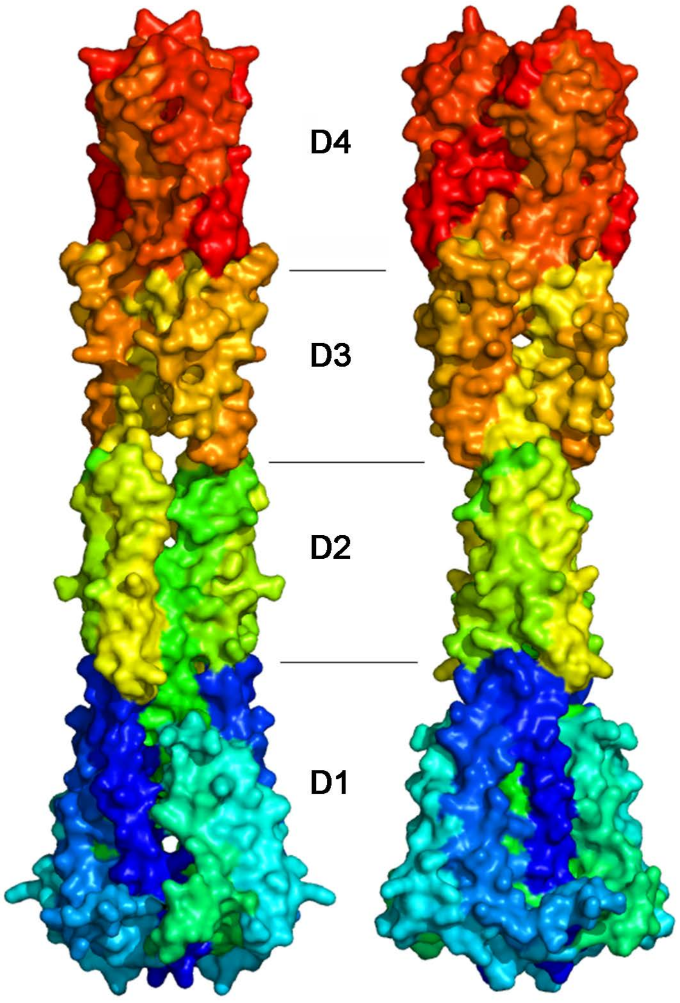

Supplementary Figure 1. Crystal structure of *Flavobacterium johnsonia* GldMp. Surface representation of GldM in two orientations at 90° from each other, around the vertical 2D axis. The colouring is in rainbow mode, from N-terminus (blue) to C-terminus (red). The four domains are numbered D1-4 from N- to C-terminus.

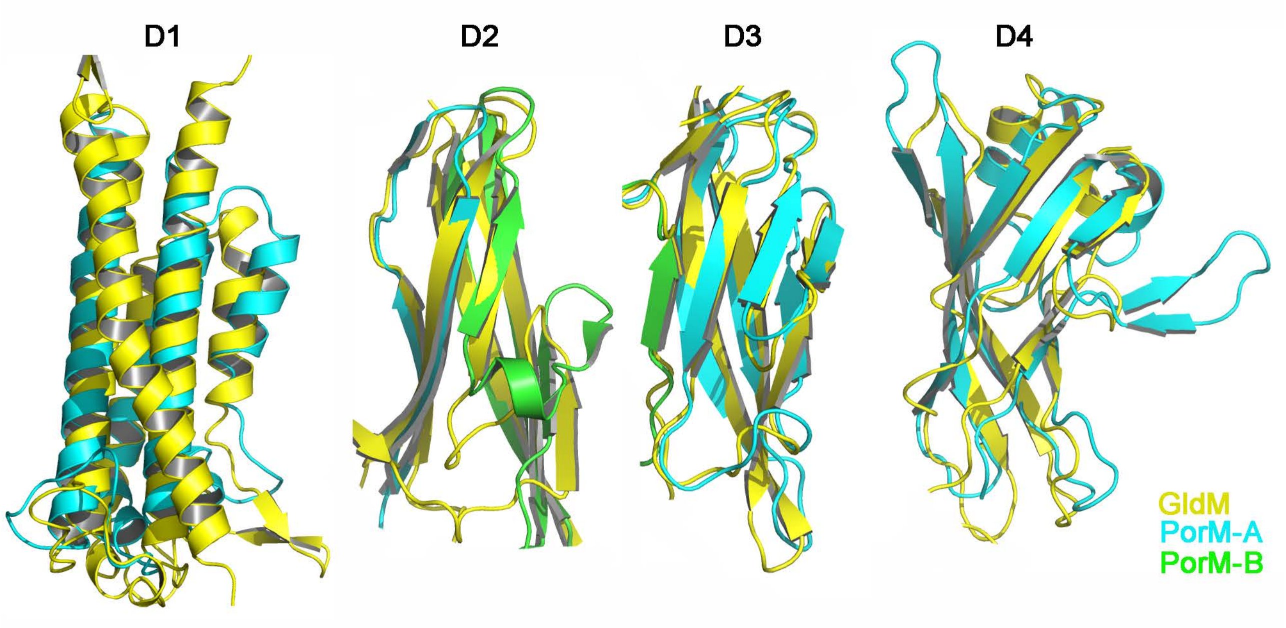

Supplementary Figure 2. Comparison of the four domains of GldMp with those of PorMp. The four domains are numbered D1-4 from N- to C-terminus. GldM domains are coloured yellow, those of PorM monomer A are colored blue. Domain swapping has been evidenced only in PorM, in which monomer B contribution to each domain is colored green.

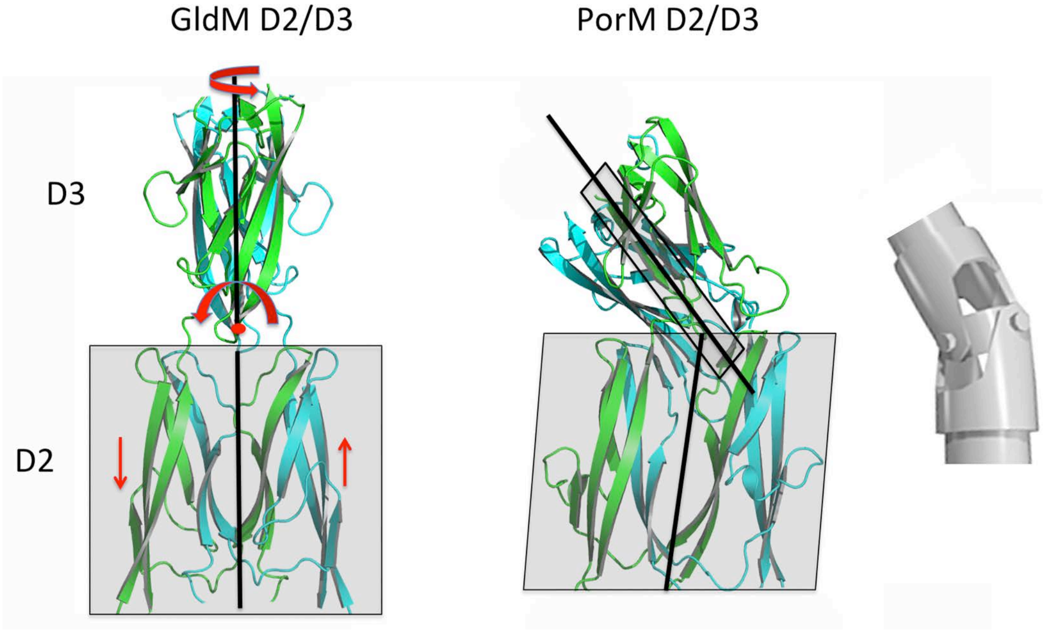

Supplementary Figure 3. Comparison of the association of domains D2 and D3 of GldM with those of PorM. Monomers A and B are coloured blue and green, respectively. The translation/rotation movements that occur to obtain the PorM D2/D3 arrangement are shown by red arrows on GldM. A view of a cardan illustrates the concept of the observed differences between GldM and PorM D2/D3 topology. Right: illustration of a universal joint created by Wapcaplet of Wikimedia Commons (https://commons.wikimedia.org/wiki/File:Universal_joint.png) in Blender, composited in the GIMP. This image is licensed under a CC-BY-SA license.

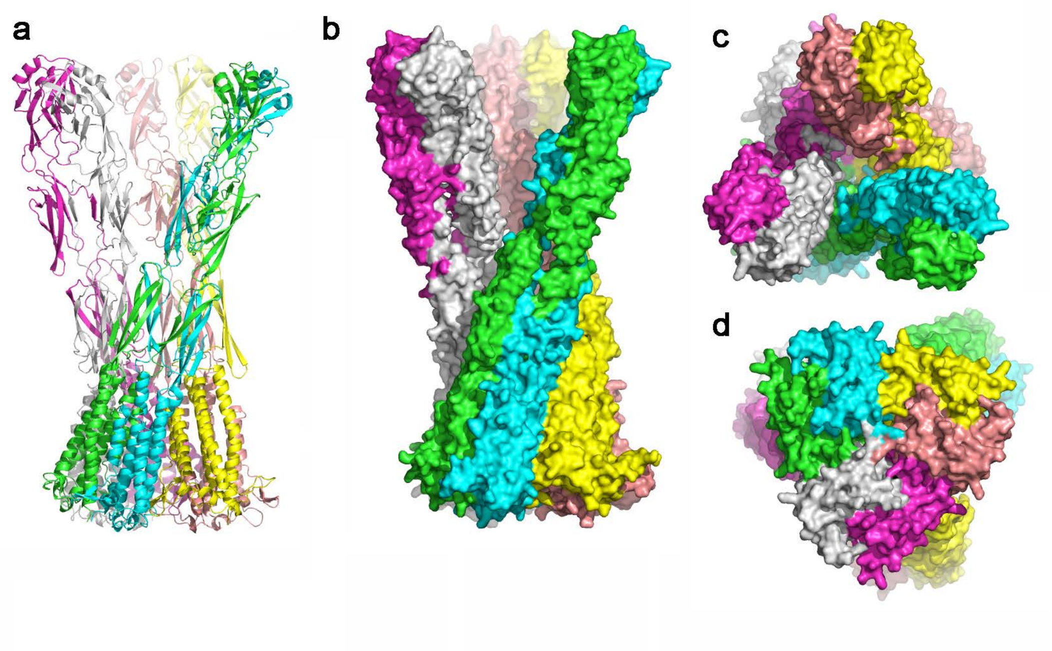

Supplementary Figure 4. Model of an arrangemment of *Flavobacterium johnsonia* GldMp dimer with 3 fold symmetry. **a**, ribbon representation of the three dimers, the six monomer being coloured individually. **b**, same view as in a, but with surface representation. **c**, same as in b, viewed from top. **d**, same as in b, viewed from bottom.

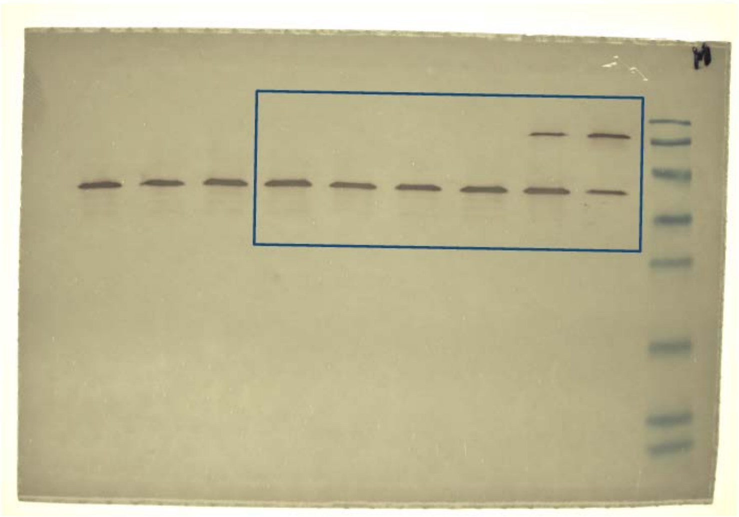

Supplementary Figure 5. Uncropped gel used in figure 4c. The part used in figure 4c is boxed blue.

Supplementary Table 1: Hydrogen and ionic bonds established between GldM monomers

Hydrogen Bonds

Monomer 1	distance(Å)	Monomer 2
A:LYS 42[NZ]	3.79	B:LYS 171[O]
A:LYS 160[NZ]	3.32	B:ASN 220[OD1]
A:LYS 170[NZ]	3.47	B:SER 49[OG]
A:LYS 180[NZ]	3.61	B:GLU 116[OE1]
A:SER 232[OG]	2.65	B:GLU 304[OE1]
A:TYR 233[N]	3.16	B:GLU 304[OE2]
A:TYR 236[OH]	2.76	B:TYR 236[OH]
A:ASN 245[ND2]	3.42	B:LYS 316[O]
A:ASN 245[ND2]	3.20	B:ASP 243[O]
A:TYR 247[N]	2.79	B:VAL 318[O]
A:GLY 250[N]	2.79	B:ALA 288[O]
A:VAL 253[N]	2.67	B:LEU 286[O]
A:GLY 255[N]	3.12	B:ILE 284[O]
A:LYS 256[NZ]	2.81	B:GLN 281[OE1]
A:LYS 256[NZ]	3.21	B:GLN 237[OE1]
A:VAL 257[N]	2.92	B:ALA 282[O]
A:ARG 261[N]	2.96	B:LEU 259[O]
A:ARG 261[NH1]	2.95	B:TYR 236[OH]
A:ALA 282[N]	3.32	B:VAL 257[O]
A:ILE 284[N]	2.81	B:GLY 255[O]
A:SER 285[OG]	3.82	B:VAL 253[O]
A:LEU 286[N]	3.27	B:VAL 253[O]
A:ALA 288[N]	2.78	B:GLU 251[O]
A:VAL 318[N]	2.80	B:ASN 245[O]
A:VAL 320[N]	2.89	B:TYR 247[O]
A:ARG 322[NE]	2.79	B:ASP 394[OD2]
A:ARG 322[NH1]	2.82	B:GLN 249[O]
A:ARG 322[NH2]	2.84	B:ASP 394[OD2]
A:ARG 322[NH2]	3.05	B:GLN 249[O]
A:ALA 326[N]	2.76	B:ASP 398[OD2]
A:THR 327[N]	2.78	B:SER 347[O]
A:THR 327[OG1]	2.79	B:THR 327[OG1]
A:SER 329[N]	2.85	B:SER 345[O]
A:ASP 331[N]	2.94	B:PRO 343[O]
A:LYS 332[NZ]	2.82	B:ASP 441[O]
A:SER 345[N]	2.76	B:SER 329[O]
A:SER 345[N]	3.41	B:ASP 331[OD1]
A:SER 345[OG]	2.73	B:ASP 331[OD1]
A:SER 347[N]	2.95	B:THR 327[O]
A:SER 347[OG]	3.81	B:THR 327[O]
A:LYS 400[NZ]	2.91	B:ILE 328[O]
A:ARG 403[NH1]	3.05	B:ASP 441[OD2]
A:ARG 403[NH2]	3.19	B:ASP 441[OD2]
A:LYS 405[NZ]	2.77	B:ASP 441[OD2]
A:LYS 405[NZ]	2.77	B:ASP 439[O]
A:THR 486[OG1]	2.67	B:GLN 484[OE1]
A:SER 49[OG]	3.47	B:LYS 170[NZ]
A:GLU 116[OE1]	3.61	B:LYS 180[NZ]
A:LYS 171[O]	3.79	B:LYS 42[NZ]
A:ASN 220[OD1]	3.32	B:LYS 160[NZ]
A:TYR 236[OH]	2.95	B:ARG 261[NH1]
A:GLN 237[OE1]	3.21	B:LYS 256[NZ]
A:ASP 243[O]	3.20	B:ASN 245[ND2]
A:ASN 245[O]	2.80	B:VAL 318[N]
A:TYR 247[O]	2.89	B:VAL 320[N]
A:GLN 249[O]	2.82	B:ARG 322[NH1]
A:GLN 249[O]	3.05	B:ARG 322[NH2]
A:GLU 251[O]	2.78	B:ALA 288[N]
A:VAL 253[O]	3.82	B:SER 285[OG]
A:VAL 253[O]	3.27	B:LEU 286[N]
A:GLY 255[O]	2.81	B:ILE 284[N]
A:VAL 257[O]	3.32	B:ALA 282[N]
A:LEU 259[O]	2.96	B:ARG 261[N]
A:GLN 281[OE1]	2.81	B:LYS 256[NZ]
A:ALA 282[O]	2.92	B:VAL 257[N]
A:ILE 284[O]	3.12	B:GLY 255[N]
A:LEU 286[O]	2.67	B:VAL 253[N]
A:ALA 288[O]	2.79	B:GLY 250[N]
A:GLU 304[OE1]	2.65	B:SER 232[OG]
A:GLU 304[OE2]	3.16	B:TYR 233[N]
A:LYS 316[O]	3.42	B:ASN 245[ND2]
A:VAL 318[O]	2.79	B:TYR 247[N]
A:THR 327[O]	3.81	B:SER 347[OG]
A:THR 327[O]	2.95	B:SER 347[N]
A:ILE 328[O]	2.91	B:LYS 400[NZ]
A:SER 329[O]	2.76	B:SER 345[N]
A:ASP 331[OD1]	2.73	B:SER 345[OG]
A:ASP 331[OD1]	3.41	B:SER 345[N]
A:PRO 343[O]	2.94	B:ASP 331[N]
A:SER 345[O]	2.85	B:SER 329[N]
A:SER 347[O]	2.78	B:THR 327[N]
A:ASP 394[OD2]	2.79	B:ARG 322[NE]
A:ASP 394[OD2]	2.84	B:ARG 322[NH2]
A:ASP 398[OD2]	2.76	B:ALA 326[N]
A:ASP 439[O]	2.77	B:LYS 405[NZ]
A:ASP 441[OD2]	2.77	B:LYS 405[NZ]
A:ASP 441[OD2]	3.05	B:ARG 403[NH1]
A:ASP 441[OD2]	3.19	B:ARG 403[NH2]
A:ASP 441[O]	2.82	B:LYS 332[NZ]
A:GLN 484[OE1]	2.67	B:THR 486[OG1]

Ionic bonds

Monomer 1	distance(Å	Monomer 2
A:LYS 42[NZ]	3.37	B:ASP 210[OD2]
A:LYS 180[NZ]	3.61	B:GLU 116[OE1]
A:ARG 322[NE]	2.79	B:ASP 394[OD2]
A:ARG 322[NH2]	2.84	B:ASP 394[OD2]
A:ARG 403[NH1]	3.41	B:ASP 441[OD1]
A:ARG 403[NH1]	3.05	B:ASP 441[OD2]
A:ARG 403[NH2]	3.19	B:ASP 441[OD2]
A:LYS 405[NZ]	3.26	B:ASP 441[OD1]
A:LYS 405[NZ]	2.77	B:ASP 441[OD2]
A:GLU 116[OE1]	3.61	B:LYS 180[NZ]
A:ASP 210[OD2]	3.37	B:LYS 42[NZ]
A:ASP 394[OD2]	2.79	B:ARG 322[NE]
A:ASP 394[OD2]	2.84	B:ARG 322[NH2]
A:ASP 441[OD1]	3.26	B:LYS 405[NZ]
A:ASP 441[OD1]	3.41	B:ARG 403[NH1]
A:ASP 441[OD2]	2.77	B:LYS 405[NZ]
A:ASP 441[OD2]	3.05	B:ARG 403[NH1]
A:ASP 441[OD2]	3.19	B:ARG 403[NH2]

Supplementary Table 2: Structural comparison of GldM and PorM domains.

Domain (monomer)	Residues in domain	Residues aligned (%)	Rmsd (Å)
D1 (α1 missing)	143	124 (86)	3.5
D2 (A+B swapped)	89	74 (83)	1.7
D3 (A+B swapped)	100	89 (89)	1.6
D4	117	104 (89)	2.15

Supplementary Table 3: oligonucleotides used in this study.

Name	Destination	Sequence (5' → 3')
For protein production [a]		
5-PorMp	insertion of *porMp* fragment into pLIC03	CCGAGAACCTGTACTTCCAATCAGATGGTTTCGACAAAGTGGATAAG
3-PorMp	insertion of *porMp* fragment into pLIC03	CGGAGCTCGAATTCGGATCCTTATTAGTTCACAATTACTTCAATGGC
5-PorMp$_{315}$	insertion of *porMp$_{315}$* fragment into pLIC03	CCGAGAACCTGTACTTCCAATCAGTGAACAGTATCACGGCACAAG
3-PorMp$_{315}$	insertion of *porMp$_{315}$* fragment into pLIC03	3-PorMp
5-PorMp$_{Nt}$	insertion of *porMp$_{Nt}$* fragment into pLIC03	CCGAGAACCTGTACTTCCAATCAAGCTTAACCTCTTCCATCGACG
5-PorMp$_{Nt}$	insertion of *porMp$_{Nt}$* fragment into pLIC03	CGGAGCTCGAATTCGGATCCTTATTAGCTCTTTACCAGATCGGCAAG
5-GldMp	insertion of *gldMp* fragment into pLIC03	CCGAGAACCTGTACTTCCAATCATTTGGTTTGATGAATGAAAAATTCG
3-GldMp	insertion of *gldMp* fragment into pLIC03	CGGAGCTCGAATTCGGATCCTTATTATTGTATTTCGTAAATTACCGG
For bacterial two-hybrid (insertion into pUT18C and pKT25) [b]		
5-BACTH-PorMp	insertion of *porMp* fragment	Vincent *et al.*, 2017
3-BACTH-PorMp	insertion of *porMp* fragment	Vincent *et al.*, 2017
3-BACTH-PorMp-D1	insertion of *porMp* D1 fragment	GAAGGGTACCCCACGATAATCCCCCACATCCACGC
5-BACTH-PorMp-D2	amplification from *porMp* D2 for insertion	GAAGTCTAGATGTGAACAGTATCACGGCACAAGTG
3-BACTH-PorMp-D3	amplification until *porMp* D3 for insertion	GAAGGGTACCCCAGCTCTGACACGTAAGGTAGTCTTTG
5-BACTH-PorMp-D4	insertion of *porMp* D4 fragment	GAAGTCTAGATCTCCCCGATCCTCTTCCCTATATCG
For site-directed mutagenesis (Cys substitutions) [c]		

A-PorM-A318C	PorM Ala318-to-Cys substitution	GGAACCAATG**TGT**TCCGTTGC
B-PorM-A318C	PorM Ala318-to-Cys substitution	GCAACGGA**ACA**CATTGGTTCC
A-PorM-A391C	PorM Ala391-to-Cys substitution	CCATCCAAATG**TGT**AAGACTACC
B-PorM-A391C	PorM Ala391-to-Cys substitution	GGTAGTCTT**ACA**CATTTGGATGG
A-PorM-M325C	PorM Met325-to-Cys substitution	GCTCCGACGATG**TGT**AATGTACTCTATG
B-PorM-A325C	PorM Met325-to-Cys substitution	CATAGAGTACATT**ACA**CATCGTCGGAGC

[a] Sequence annealing on the target plasmid underlined.
[b] Restriction site underlined.
[c] Mutagenized codon **in bold**.
